# Gills are not the primary site of gas exchange in the suspension-feeding hemichordate *Protoglossus graveolens*

**DOI:** 10.1242/jeb.253087

**Published:** 2026-09-11

**Authors:** Michael A. Sackville, Christopher B. Cameron, Colin J. Brauner

**Affiliations:** ^1^ Department of Biology, University of Ottawa, Ottawa, ON, Canada, K1N 6N5; ^2^ Département de Sciences Biologiques, Université de Montréal, Montréal, PQ, Canada, H3C 3J7; ^3^ Department of Zoology, University of British Columbia, Vancouver, BC, Canada, V6T 1Z4

**Keywords:** Deuterostome, Pharyngeal gill slits, Vertebrate evolution

## Abstract

The gills are hypothesized to play a key role in early vertebrate evolution by replacing the skin as the primary site of gas exchange. In this scenario, water flow across the gills for suspension feeding was coopted for gas exchange in stem vertebrates, facilitating the evolution of larger, active fishes. This is supported by a stem-vertebrate origin for structures increasing gill capacity for gas exchange. However, these structures might have enhanced an already dominant capacity at ancestral deuterostome gills. To test this, we characterized gill function in the suspension-feeding hemichordate *Protoglossus graveolens*. We measured oxygen uptake and ammonia excretion in whole worms and worm halves with or without gills at 10°C and 20°C to stimulate gill recruitment. Gills did not enhance oxygen uptake or ammonia excretion, suggesting they are not a primary site of gas exchange. This provides critical functional support for the long-hypothesized vertebrate origin of gills as the primary gas exchange organ.

## Teaser

**Highlighted Article:** New functional data from hemichordates provide critical support for a vertebrate origin of gas exchange at gills.

## INTRODUCTION

The origin and evolution of early vertebrates is characterized by a transition from small, worm-like organisms to larger, more active fishes (Cameron et al., 2000). The gills are hypothesized to play a key role in this iconic transition by replacing the skin as the primary site of gas exchange (Gans and Northcutt, 1983; Northcutt, 2005; Brauner and Rombough, 2012). In this scenario, ancestral deuterostomes were constrained to worm-like body forms and activity by relying on the skin for gas exchange. This reliance required a high body surface area to volume ratio and thin dermis to enable sufficient gas diffusion. Water flow across the gills used to filter feed by ancestral deuterostomes was then exploited and enhanced for gas exchange by pharyngeal adaptations in stem vertebrates. This relaxed the worm-like constraints on body form and activity imposed by gas exchange at the skin, thereby facilitating the evolution of larger and more active fishes.

Support for this scenario is compelling but incomplete. Fossil and developmental studies support a stem-vertebrate origin for structures that increase gill capacity for gas exchange, including a muscular pharyngeal pump, lamellar projections and pillar cells (Shu et al., 1999; Xian-guang et al., 2002; Mongera et al., 2013; Green and Bronner, 2014; Morris and Caron, 2014; Green et al., 2015; Gillis and Tidswell, 2017). However, it is unknown whether these structures mark a true shift from skin to gills for gas exchange, or whether they simply enhance an already dominant capacity at the gills. *In vivo* measurements in the hemichordate *Saccoglossus kowalevskii* suggest that gills are not the primary site of gas exchange in this key invertebrate outgroup (Sackville et al., 2022). However, this is the only invertebrate deuterostome in which gill function for gas exchange has been tested. Moreover, aspects of *S. kowalevskii*’s feeding mode suggest that it might be expected to have a lower capacity for gas exchange at gills than other outgroup members.

Indeed, *S. kowalevskii* does not rely on pharyngeal water flow to feed like the proposed vertebrate ancestor and many other invertebrate deuterostomes (Cameron et al., 2000; Cameron, 2005; Lowe et al., 2015; Simakov et al., 2015; Nanglu et al., 2023; Li et al., 2023). *Saccoglossus* is a deposit-feeding filter feeder, which instead collects benthic sediment externally with its long, mucus-covered proboscis (Miller, 1992; Gonzalez and Cameron, 2009). Collected sediment is then physically passed from the proboscis into the pharynx through the mouth by cilia. Once in the pharynx, intermittent contractions form a concentrated mucus-food cord for ingestion, with excess fluid leaving through the gill slits. This differs from suspension-feeding filter feeders, where large amounts of water flow into the pharynx and out through the gill slits by way of a cilia-driven ‘inspiratory’ current. Here, the inspiratory current transports fine food particles from the water column into the pharynx, where they become captured by the mucociliary epithelium of the pharyngeal gill bars (Gonzalez and Cameron, 2009). Because the deposit-feeding *S. kowalevskii* does not rely on an inspiratory current to feed in this way, it may lack the ability to generate sufficient water flow or ‘ventilation’ through the pharynx to breathe with gills relative to suspension feeders. The finding that gills do not play a primary role in gas exchange in *S. kowalevskii* might therefore be a specialized trait linked to deposit feeding rather than a general representation of gill function in this outgroup. More robust support for a vertebrate origin of gas exchange at the gills thus requires further investigation in more invertebrate deuterostomes, and preferably those that suspension feed rather than deposit feed.

Here, we addressed this knowledge gap by testing gill function for gas exchange in the hemichordate *Protoglossus graveolens*. *Protoglossus graveolens* closely resembles the morphology and ecology of *S. kowalevskii*, but suspension feeds (Gonzalez and Cameron, 2009) and has a body size that is twice as large [mean wet mass of 0.236 g (*P. graveolens*; present study) versus 0.105 g (*S. kowalevskii*; Sackville et al., 2022)]. We hypothesized that this suspension feeding machinery and larger body size facilitate and increase gill recruitment for gas exchange. To test this hypothesis, we leveraged the regenerative capacity of *P. graveolens* to separate worms into viable halves with and without gills for respirometry as done for *S. kowalevskii* (Sackville et al., 2022). This approach is supported by the widespread occurrence of regeneration in hemichordates, where worm fragments typically regenerate and/or persist long after separation (Tweedell, 1961; Rychel and Swalla, 2009; Humphreys et al., 2010). We took measurements at 10°C and during an acute thermal challenge at 20°C to stimulate maximum gill recruitment. We predicted that worm halves without gills would have a reduced capacity for gas exchange relative to whole worms and worm halves with gills, and would therefore have lower mass-specific rates of oxygen uptake and ammonia excretion.

## MATERIALS AND METHODS

### Animal husbandry

*Protoglossus graveolens* Giray and King 1996 were collected from Lowe's Cove near the Darling Marine Center in Walpole, ME, USA, and shipped to the University of British Columbia's Point Grey campus in Vancouver, Canada. Worms were held at 10°C on a 12 h:12 h light:dark photoperiod in static seawater tanks with sandy substrate (10 worms per 40 liter tank; 34 ppt artificial seawater made from deionized RO water and Instant Ocean salt mix, Instant Ocean, USA). Worms had a mean wet mass of 0.236±0.021 g (*n*=27) and were acclimated for 5 days before experimentation.

### Ethics

All animal care and experimentation conformed to the guidelines set by the Canadian Council on Animal Care (CCAC) and were approved by the University of British Columbia's Animal Care Committee (ACC) under the animal use protocol A19-0284.

### 10°C protocol

Seven whole worms were placed in custom acrylic respirometers (11 ml) in an aerated water bath at 10°C. Each respirometer had a needle-type oxygen probe (Presens, Germany), overflow tube and a stir bar physically separated from worms by a fine mesh grate. Respirometers were left open for 1 h to acclimate worms, then sealed to record oxygen partial pressure (*P*_O_2__; mm Hg) over ∼1–3 h. Worms were then removed from respirometers, cut in half with a transverse section posterior to the gill pores, and moved to 10 ml aerated chambers at 10°C to recover overnight. Once recovered, worm halves were returned to separate respirometers to record *P*_O_2__ as before. Following these recordings, worm halves were placed in 20 ml aerated chambers where 1 ml water samples were taken at 10 min and 12 h. Water samples were frozen at −20°C for later measurement of total ammonia as described below. Six additional naive worms were also placed in 20 ml chambers for measurement of ammonia excretion in whole animals. At trial completion, all worms were euthanized with a lethal dose of MS-222 and weighed. Wet masses for whole worms were calculated from the sums of corresponding worm halves. Whole worms were not weighed prior to transection because they are unlikely to survive emersion and handling. Ammonia excretion was not measured during the respirometry trials because reliable detection required incubation times of up to 12 h in some cases.

### 20°C protocol

Eight whole worms were warmed from 10°C to 20°C overnight at 2.5°C h^−1^ in a water bath. Worms were then subjected to the same protocol as outlined above for 10°C, but at 20°C. A temperature of 20°C was chosen as an ecologically relevant thermal challenge that would significantly increase oxygen demand from 10°C, but not result in mortality over 24 h. This temperature was chosen based on prior work with the hemichordate *S. kowalevskii* that recommended rearing temperatures not exceed 24°C (Lowe et al., 2004).

### O_2_ uptake

*P*_O_2__ was recorded from each respirometer with OXY-4V2_11TX data acquisition software (Presens). Mass-specific oxygen uptake rates (*Ṁ*_O_2__; μmol O_2_ g^−1^ h^−1^) were then calculated from linear declines in *P*_O_2__ with the following equation:

(1) $${\dot{M}}_{O_{2}}=\alpha \Delta P_{O_{2}}(V_{r}-V_{w})t^{-1}m^{-1},$$

where α is oxygen solubility (1.7934 and 1.4842 μmol O_2_ l^−1^ mmHg^−1^ at 10°C and 20°C, respectively; Boutilier et al., 1984), *V*_r_ is respirometer volume (l), *V*_w_ is worm volume (l; assuming neutral buoyancy where 1 l=1 kg), *t* is time (h) and *m* is worm mass (g). Background respiration was negligible, which was aided by respirometer disinfection (Virkon; Antec International, UK) and the preparation of fresh seawater with deionized RO water and Instant Ocean salt mix before each trial.

To accommodate the unusual morphology of *P. graveolens* while maintaining good water mixing in respirometers, the respirometer:organism volume ratios were often larger than the recommended 20–50:1 for fishes (Svendsen et al., 2016). These larger ratios resulted in slower declines in chamber *P*_O_2__, requiring 0.5–2.0 h for a 10% drop in air saturation depending on water temperature and worm/fragment mass. To keep trial durations at manageable lengths, only single traces were recorded from each worm/fragment. Each trace (1) spanned at least a 10% drop in air saturation, (2) was recorded between 80% and 100% air saturation and (3) had an *r*^2^≥0.95.

### NH_3/4_^+^ excretion

Water samples were analyzed for total ammonia (NH_3/4_^+^) colourometrically (Verdouw et al., 1978) in triplicate using a SpectraMax 190 microplate reader (Molecular Devices, USA) as in Sackville et al. (2022). The difference in ammonia concentration over trial duration (ΔNH_3/4_^+^; μmol NH_3/4_^+^ ml^−1^) was used to calculate ammonia excretion rates (*Ṁ*_NH_3/4_^+^_; μmol NH_3/4_^+^ g^−1^ h^−1^) with the following equation:

(2) $${\dot{M}}_{NH_{3/4}^{+}}=\Delta NH_{3/4}^{+}(V_{r}-V_{w})m^{-1}t^{-1},$$

where *V*_r_ is respirometer volume (l), *V*_w_ is worm volume (l; assuming neutral buoyancy where 1 l=1 kg), *m* is worm mass (g) and *t* is time (h).

As outlined in the experimental protocol, excretion rates for whole worms were measured in separate naive animals rather than in those later subjected to fragmentation. This was done at 20°C to reduce time spent in the acute thermal challenge before fragmentation by 12 h, limiting the potential for acclimation between measurements in whole and fragmented worms. The same was also done at 10°C for consistency across treatments.

### Statistics

Data were analyzed with Prism 11 for macOS (Version 9.3.1; GraphPad Software Inc., USA). Oxygen uptake in whole and fragmented worms was analyzed with one-way repeated measures ANOVA with Geisser–Greenhouse correction and Tukey's *post hoc* test. Ammonia excretion was analyzed with ordinary one-way ANOVA and Tukey's *post hoc* test. All data passed tests of normality and equal variance, but some required transformation [1/*x* for *Ṁ*_O_2__ and *Ṁ*_NH_3/4_^+^_ at 10°C; log(*x*) for *Ṁ*_NH_3/4_^+^_ at 20°C].

Animals were randomly assigned to experimental treatments, but blinding was not possible as water temperature required monitoring and manipulation during experiments. All other data collection and analyses were blind to treatments.

## RESULTS AND DISCUSSION

**Fig. 1.**
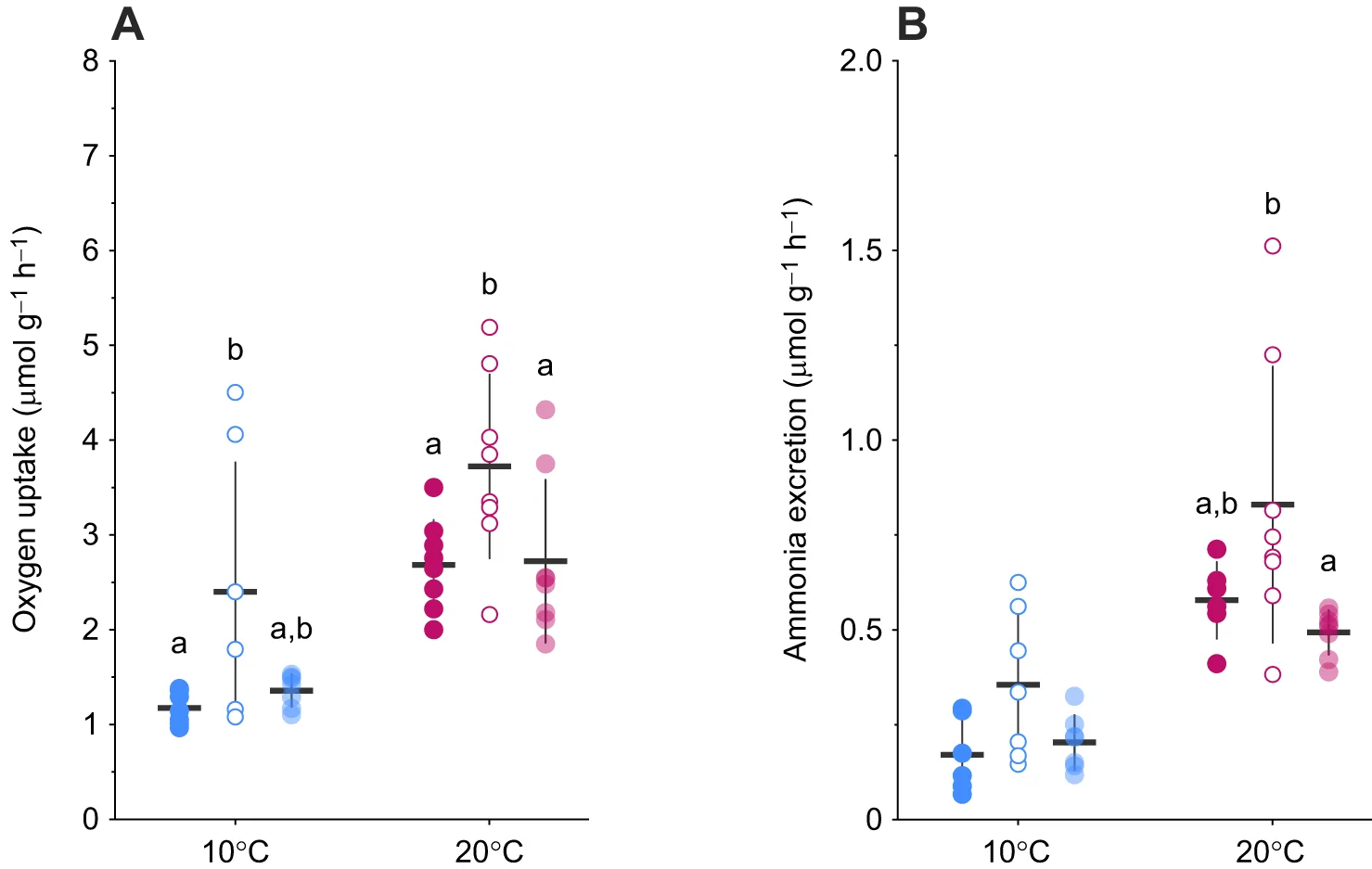
**Gill presence is not associated with elevated rates of gas exchange in *Protoglossus graveolens*.** Mass-specific rates of (A) oxygen uptake and (B) ammonia excretion in whole worms (solid symbols), worm halves without gills (open symbols) and worm halves with gills (shaded symbols) at 10°C (blue) and 20°C (red). Data are presented as means±s.d. with individuals superimposed. Letters that differ indicate significant differences within treatments (one-way repeated measures ANOVA with Tukey's test for oxygen uptake, *P*<0.05, *n*=7 for 10°C, *n*=8 for 20°C; one-way ANOVA with Tukey's test for ammonia excretion, *P*<0.05, *n*=6 for whole worms, *n*=7 for halves at 10°C, *n*=8 for halves at 20°C).

Our results indicate that gills are not the primary site of gas exchange in the suspension-feeding hemichordate *P. graveolens*. Mass-specific rates of oxygen uptake (*Ṁ*_O_2__) and ammonia excretion (*Ṁ*_NH_3/4_^+^_) for worm halves without gills were at least as high as rates for whole worms and worm halves with gills (Fig. 1A,B). This was true at both temperatures despite a 1.9- and 2.6-fold increase at 20°C for *Ṁ*_O_2__ and *Ṁ*_NH_3/4_^+^_, respectively. Rates were similar to those reported for the deposit-feeding hemichordate *S. kowalevskii*, suggesting that our experimental preparation did not affect worms in any unexpected way (Sackville et al., 2022). Furthermore, temperature effects (*Q*_10_; ratio between rates over a span of 10°C) lower than 2 for *Ṁ*_O_2__ and above 2.5 for *Ṁ*_NH_3/4_^+^_ support 20°C as a suitable challenge near an upper thermal limit (Schulte, 2015).

**Fig. 2.**
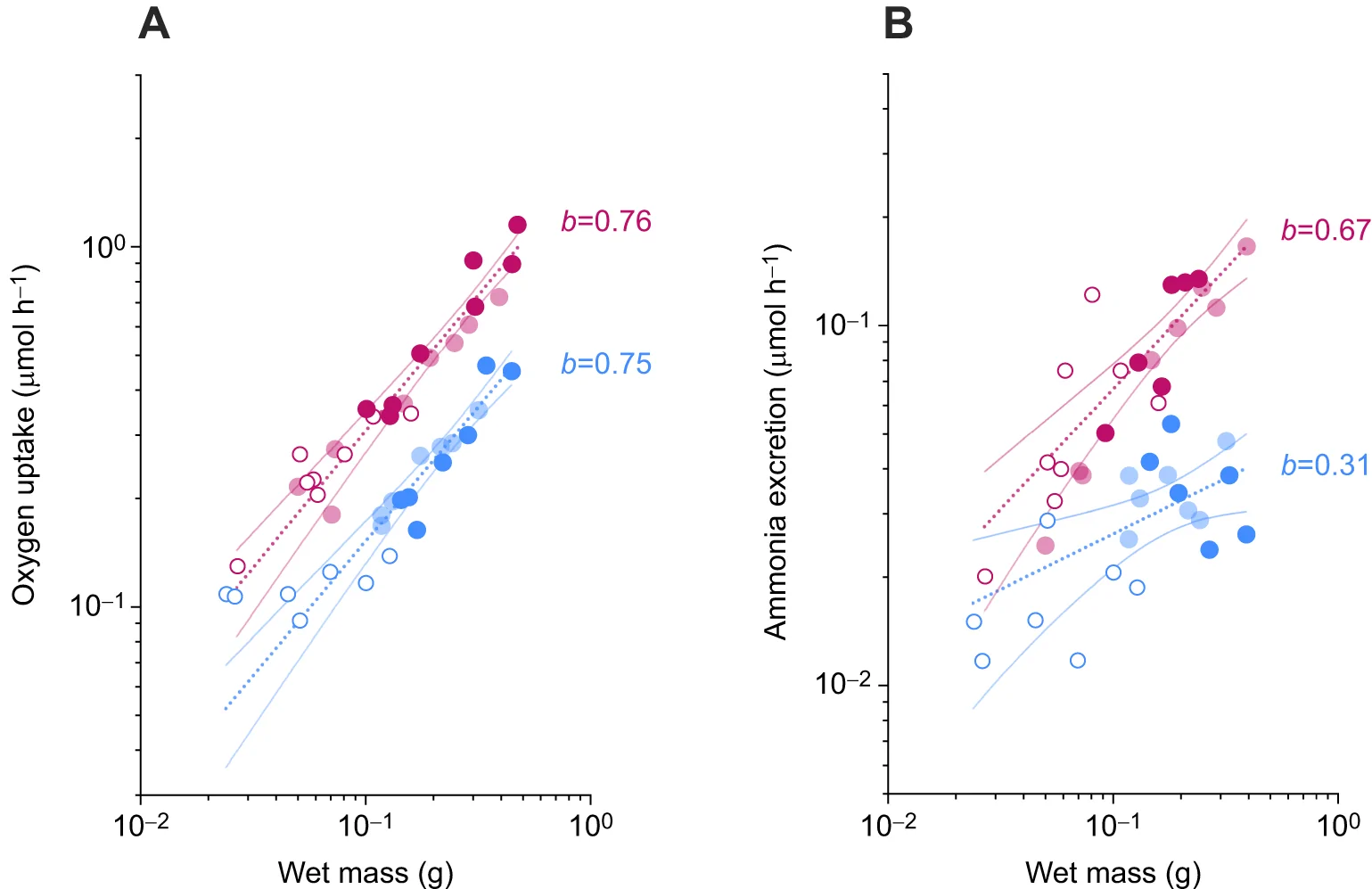
**Rates of gas exchange scale with worm and fragment mass.** Rates of (A) oxygen uptake and (B) ammonia excretion expressed as a function of wet mass in whole worms (solid symbols), worm halves without gills (open symbols) and worm halves with gills (shaded symbols) at 10°C (blue) and 20°C (red). Allometric curves ±95% CI are fitted to data for each temperature. Oxygen uptake at 10°C (*b*=0.75, *r*^2^=0.90, *n*=21) and 20°C (*b*=0.76, *r*^2^=0.92, *n*=24); ammonia excretion at 10°C (*b*=0.31, *r*^2^=0.36, *n*=20) and 20°C (*b*=0.67, *r*^2^=0.74, *n*=22).

In some treatments, worm halves without gills had a higher *Ṁ*_O_2__ and *Ṁ*_NH_3/4_^+^_ than whole worms or worm halves with gills (*Ṁ*_O_2__ at 10°C and 20°C, *Ṁ*_NH_3/4_^+^_ at 20°C; Fig. 1). This appears to be associated with differences in worm fragment mass (Fig. 2). Indeed, oxygen uptake scales with fragment mass to the power of approximately 3/4 at both temperatures (Fig. 2A), and ammonia excretion to the power of approximately 2/3 at 20°C (Fig. 2B). This results in higher mass-specific rates for worm fragments without gills as they are generally smaller than whole worms and fragments with gills. Moreover, when corrected for scaling (Supplementary Materials and Methods), these rates no longer differ significantly (Fig. S1). Determining the underlying cause of this scaling relationship is beyond the scope of our work, but we suspect that differences in worm or fragment surface area to volume ratio might be affecting rates of cutaneous gas exchange. Future experiments using larger samples sizes and morphometric measurements can test this idea more rigorously and help determine whether other, more complex factors might also be at play.

The similar rates of gas exchange between whole worms and worm fragments of similar size suggest that the circulatory system in general likely plays a minor role in oxygen uptake and ammonia excretion, as fragmentation would interrupt continuity. This is perhaps unsurprising given that hemichordate hemolymph lacks an oxygen carrying pigment and their open circulation has low convective power (Barrington, 1965). Worms do possess a muscular sac-like heart in the proboscis that may aid circulation, but most hemolymph movement is likely driven by muscular contractions of the body wall during locomotion (Barrington, 1965). This apparent lack of reliance on a centralized exchange organ and circulatory system for gas exchange may benefit survival during fragmentation, with wounds reportedly taking 2–3 days to heal over with new body wall (Tweedell, 1961; Humphreys et al., 2010). To our knowledge, natural fragmentation rates for hemichordates by predation and/or wave damage in the wild have yet to be quantified, but it could be an important selective pressure limiting gill and circulatory recruitment and adaptation for gas exchange in these animals.

This is the first test of gill function for gas exchange in a suspension-feeding invertebrate deuterostome, and only the second invertebrate deuterostome to be tested at all. Together with prior findings in *S. kowalevskii* (Sackville et al., 2022), these results suggest that the gills of hemichordate acorn worms do not play a significant role in gas exchange regardless of whether they suspension feed or deposit feed. This is essential and compelling support for a vertebrate origin of gas exchange at gills, as these data are the only functional measurements from this key outgroup. However, additional measurements in *P. graveolens* and other taxa would help strengthen these conclusions. In *P. graveolens*, we were unable to monitor the inspiratory current in our respirometers, and the experimental conditions could have reduced this water current and, potentially, gill recruitment for gas exchange. Although challenging, future experiments using flow-through respirometry and water dye could measure inspiratory current and gas exchange simultaneously to test this possibility. Measurements should also be made during hypoxia, as done for *S. kowalevskii* (Sackville et al., 2022), as the gills might be preferentially recruited for gas exchange during this ecologically relevant challenge. Beyond hemichordates, functional measurements should also be made in other key outgroups within the deuterostomes that possess homologous pharyngeal gill slits, including the cephalochordates and urochordates. These taxa are more closely related to vertebrates than hemichordates and should be similarly targeted for future study. Although neither of these taxa possess respiratory pigments or circulatory characteristics that indicate a reliance on gills for gas exchange (Macara et al., 1979; Rahr, 1981), we believe that functional studies are still a worthy endeavour. Functional measurements that show the absence of a primary role for gills in gas exchange in these other taxa will allow us to more confidently resolve the origin of breathing at gills to the vertebrate stem, and to determine whether the pattern observed in hemichordate acorn worms truly represents a shared ancestral condition among all invertebrate deuterostomes.

## Supplementary Material

10.1242/jexbio.253087_sup1 Supplementary information
