# Supplementary material for: Gills are not the primary site of gas exchange in the suspension-feeding hemichordate Protoglossus graveolens
Source: J Exp Biol. 2026 Sep 11;229(17):jeb253087. doi: 10.1242/jeb.253087 (PMC13615691; doi:10.1242/jeb.253087)
Supplement: Supplementary file 1 [file jexbio-229-253087-s1.pdf]

Supplementary Materials and Methods

To correct for differences in worm/fragment mass, we expressed oxygen consumption and ammonia excretion rates as a function of worm/fragment mass raised to the power of their mass exponents (*b*). These data are presented in Figure S1 as µmols of O_2 or $NH_{3/4}^+ \cdot h^{-1} \cdot g^{-b}$. Mass exponents for each treatment and gas were calculated in Figure 2 of the main text and, when used in this way, provide mass-independent rates (Brauner and Czech, 2011).

Data were analyzed with Prism 11 for macOS (Version 9.3.1; GraphPad Software Inc., USA). Oxygen uptake in whole and fragmented worms was analyzed with one-way repeated measures ANOVA. A Geisser-Greenhouse correction and Holm-Sidak post-hoc test were applied to $\dot{M}O_2$ at 10°C, and Tukey's post-hoc test was applied to $\dot{M}O_2$ at 20°C. Ammonia excretion was analyzed with ordinary one-way ANOVA and Tukey's post-hoc test. Except for $\dot{M}O_2$ at 10°C, all data passed tests of normality and equal variance. However, $\dot{M}NH_{3/4}^+$ at 10°C required transformation (1/x).

References

Cech, J. J. and Brauner, C. J. (2011). Techniques in whole animal respiratory physiology. In *Encyclopedia of Fish Physiology* (ed. A. P. Farrell), pp. 845-853. San Diego: Academic Press

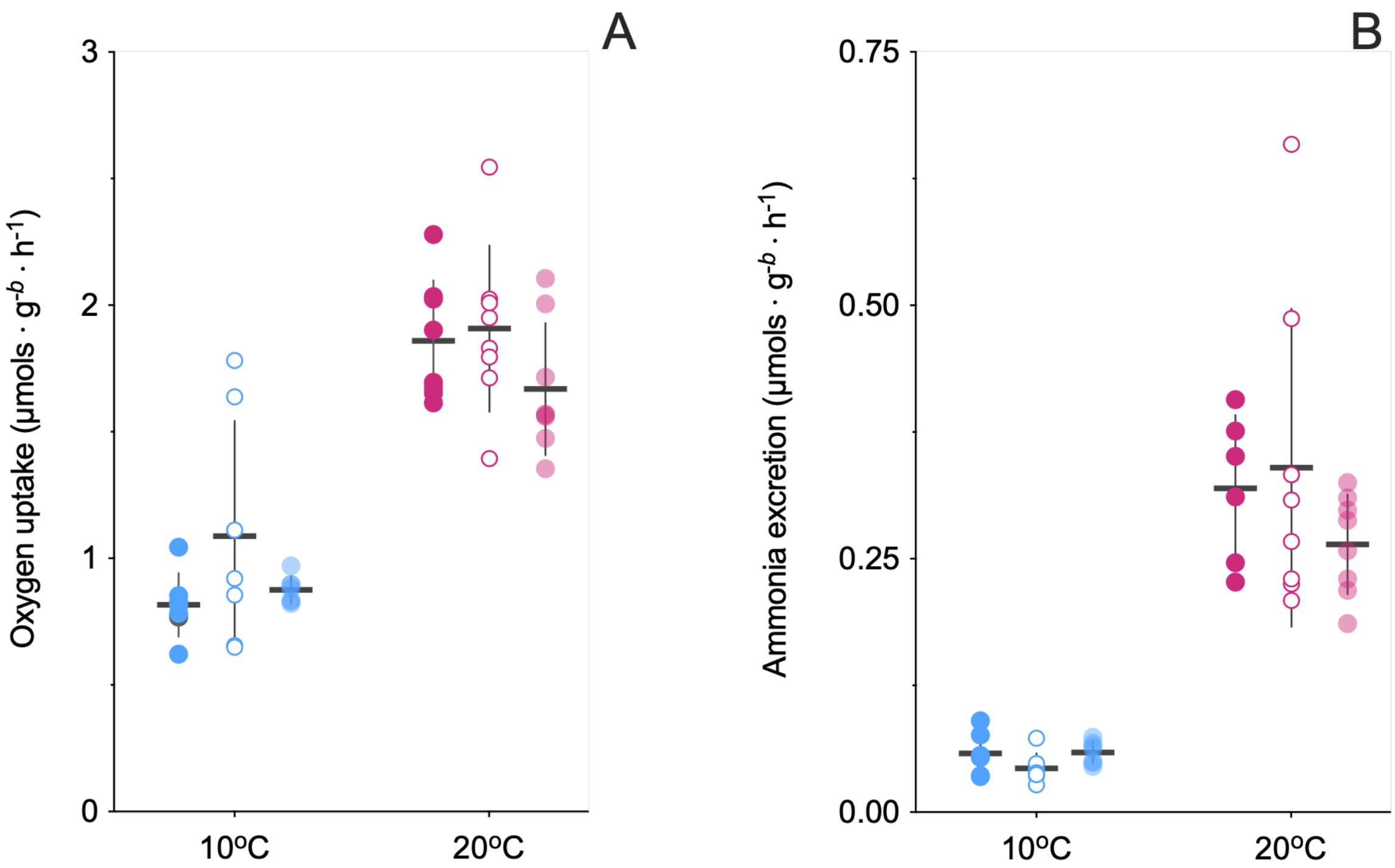

Fig. S1. Mass-independent rates of (A) oxygen uptake and (B) ammonia excretion in whole worms (solid symbols), worm halves without gills (open symbols) and worm halves with gills (shaded symbols) at 10ºC (blue) and 20ºC (red). Data presented as means±s.d. with individuals superimposed. One-way repeated measures ANOVA with Holm-Sidak and Tukey's tests for oxygen uptake at 10ºC and 20ºC, respectively (P<0.05, n=7 for 10ºC, n=8 for 20ºC). One-way ANOVA with Tukey's test for ammonia excretion (P<0.05, n=6 for whole worms, n=7 for halves at 10ºC, n=8 for halves at 20ºC). Mass exponents (b) for oxygen uptake (0.75 at 10ºC; 0.76 at 20ºC) and ammonia excretion (0.31 at 10ºC; 0.67 at 20ºC).

Journal of Experimental Biology • Supplementary information
